# Identification, Heterologous Expression, and Functional Characterization of *Bacillus subtilis* YutF, a HAD Superfamily 5'-Nucleotidase with Broad Substrate Specificity

**DOI:** 10.1371/journal.pone.0167580

**Published:** 2016-12-01

**Authors:** Natalia P. Zakataeva, Dmitriy V. Romanenkov, Yuliya R. Yusupova, Victoria S. Skripnikova, Takayuki Asahara, Sergey V. Gronskiy

**Affiliations:** 1 Ajinomoto-Genetika Research Institute, Moscow, Russia; 2 Research Institute for Bioscience Products & Fine Chemicals, Ajinomoto Co., Inc, Kawasaki, Kanagawa, Japan; Universidade Nova de Lisboa, PORTUGAL

## Abstract

5'-nucleotidases (EC 3.1.3.5) catalyze the hydrolytic dephosphorylation of 5'-ribonucleotides and 5'-deoxyribonucleotides as well as complex nucleotides, such as uridine 5'-diphosphoglucose (UDP-glucose), nicotinamide adenine dinucleotide and flavin adenine dinucleotide, to their corresponding nucleosides plus phosphate. These enzymes have been found in diverse species in intracellular and membrane-bound, surface-localized forms. Soluble forms of 5'-nucleotidases belong to the ubiquitous haloacid dehalogenase superfamily (HADSF) and have been shown to be involved in the regulation of nucleotide, nucleoside and nicotinamide adenine dinucleotide (NAD^+^) pools. Despite the important role of 5'-nucleotidases in cellular metabolism, only a few of these enzymes have been characterized in the Gram-positive bacterium *Bacillus subtilis*, the workhorse industrial microorganism included in the Food and Drug Administration’s GRAS (generally regarded as safe) list. In the present study, we report the identification of a novel 5'-nucleotidase gene from *B*. *subtilis*, *yutF*, which comprises 771 bp encoding a 256-amino-acid protein belonging to the IIA subfamily of the HADSF. The gene product is responsible for the major *p*-nitrophenyl phosphatase activity in *B*. *subtilis*. The *yutF* gene was overexpressed in *Escherichia coli*, and its product fused to a polyhistidine tag was purified and biochemically characterized as a soluble 5'-nucleotidase with broad substrate specificity. The recombinant YutF protein was found to hydrolyze various purine and pyrimidine 5'-nucleotides, showing preference for 5'-nucleoside monophosphates and, specifically, 5'-XMP. Recombinant YutF also exhibited phosphohydrolase activity toward nucleotide precursors, ribose-5-phosphate and 5-phosphoribosyl-1-pyrophosphate. Determination of the kinetic parameters of the enzyme revealed a low substrate specificity (*K*_*m*_ values in the mM concentration range) and modest catalytic efficiencies with respect to substrates. An initial study of the regulation of *yutF* expression showed that the *yutF* gene is a component of the *yutDEF* transcription unit and that YutF overproduction positively influences *yutDEF* expression.

## Introduction

Nucleotidases are enzymes that catalyze the hydrolytic dephosphorylation of nucleotides to nucleosides and phosphates. 5'-nucleotidases (EC 3.1.3.5) cleave the phosphate from the 5' end of the sugar moiety and hydrolyze 5'-ribonucleotides and 5'-deoxyribonucleotides as well as complex nucleotides, such as uridine 5'-diphosphoglucose (UDP-glucose), nicotinamide adenine dinucleotide and flavin adenine dinucleotide. These enzymes are widely distributed among all domains of life [1]. Various 5'-nucleotidases differ with respect to their range of hydrolyzed substrates and exist in intracellular or in membrane-bound, surface-localized forms. The physiological functions of 5'-nucleotidases depend on their cellular localization and differ in various organisms and tissues. Most of the well-studied 5'-nucleotidases from eukaryotes have been shown to be involved in purine and pyrimidine salvage pathways, nucleic acid repair, cell-to-cell communication, and signal transduction, among others. The 5'-nucleotidases, together with nucleoside kinases, regulate the cellular concentration of ribo- and deoxyribonucleoside monophosphates and, therefore, control the ribo- and deoxyribonucleotide pools [2–4].

In contrast to the well-studied mammalian nucleotidases, only a few 5'-nucleotidases from bacteria have been cloned and characterized. The periplasmic bifunctional UDP-sugar hydrolase/5'-nucleotidase UshA from *Escherichia coli*, which is homologous to the mammalian ecto-5'-nucleotidases, has been shown to have important functions in nucleotide salvage and to be required for growth on 5'-AMP as a sole carbon source [5,6]. Recently, a key role has been shown for this enzyme in NAD degradation [7]. Protein homologs of *E*. *coli* UshA have been identified and studied in *Corynebacterium glutamicum* and *Bacillus subtilis* [8,9]. UshA from *C*. *glutamicum* is a secreted enzyme that possesses UDP-sugar hydrolase and 5'-nucleotidase activities and allows the growth of cells on nucleotides as a carbon source. UshA is an important component of the phosphate starvation response in *C*. *glutamicum* [8]. The extracellular protein YfkN from *B*. *subtilis* exhibits 2',3'-cyclic nucleotide 2'-phosphodiesterase, 2' (or 3')-nucleotidase and 5'-nucleotidase activities and plays an important role in the recovery of inorganic phosphate and in the regulation of intercellular signaling [9].

Most of the soluble intracellular 5'-nucleotidases from humans, yeasts and bacteria [10–13] belong to the vast haloacid dehalogenase superfamily (HADSF), which includes enzymes that use an active site aspartate involved in nucleophilic catalysis to catalyze carbon or phosphoryl group transfer reactions on a diverse range of substrates [14]. Several HADSF members have been identified and characterized in *E*. *coli* as multifunctional enzymes that exhibit remarkably broad and overlapping substrate spectra [11]. One of these enzymes, UmpH (NagD), can recognize deoxyribo- and ribonucleoside tri-, di- and monophosphates as well as phosphates, polyphosphate and glucose-1-P as substrates [11], demonstrating the highest specificity for the nucleoside monophosphates, UMP and GMP [10,15]. UmpH belongs to COG0647 (ribonucleotide monophosphatase NagD, HAD superfamily, ftp://ftp.ncbi.nih.gov/pub/COG/COG2014/static/byCOG/COG0647.html), which includes two representatives from *B*. *subtilis*, AraL and YutF. AraL has been previously characterized as a sugar phosphatase with a low specificity toward several sugar phosphates, which are metabolic intermediates of the glycolytic and pentose phosphate pathways [16]. YutF is an uncharacterized protein, which was annotated in the NCBI protein database (http://www.ncbi.nlm.nih.gov/protein) as a putative hydrolase or *p*-nitrophenyl phosphatase (*p*NPPase). In the present study, we report the molecular cloning, heterologous expression, purification and functional characterization of YutF. This protein has been characterized as a 5'-nucleotidase with phosphohydrolase activity toward a number of substrates. The enzyme catalyzes the dephosphorylation of the non-natural substrate, *p*-nitrophenyl phosphate (*p*NPP), and various purine and pyrimidine 5'-nucleotides, exhibiting the highest catalytic activity toward 5'-XMP. Moreover, YutF can also recognize 5-phosphoribosyl-1-pyrophosphate (PRPP) and ribose-5-phosphate (R5P) as substrates. We also present the initial study of *yutF* expression in the context of the *yutDEF* operon.

## Materials and Methods

### Bacterial strains and plasmids

The bacterial strains and plasmids used in this study are listed in Table 1. *E*. *coli* was used as a host for cloning and protein expression. All *B*. *subtilis* strains were constructed using the delivery plasmids as indicated in Table 1. When pNZT1 derivatives were used as the delivery plasmids, a two-step replacement recombination procedure was applied to obtain the recombinant strains [17]. Strains constructed using the pMUTIN2- or pDG268-based plasmids were selected as single-crossover or double-crossover chromosomal integrants, respectively, using antibiotic selection. The single-crossover was maintained by erythromycin resistance. The primers used in this study are shown in Table 2.

**Table 1:** Bacteria and plasmids used in this study.

Strain or plasmid	Relevant characteristics^a^	Source or description^b^
*Escherichia coli* strains		
TG1	*supE hsdΔ5 thi Δ*(*lac-proAB*)*/*F' *traΔ*36 *proA*^*+*^*B*^*+*^ *lacI*^*q*^ *lacZΔ*M15	VKPM B5837
BL21(DE3)	Host for pET vectors. λDE3, *ompT*	Novagen
*Bacillus subtilis* strains		
168	*trpC2*	VKPM B1727 [18]
Bs*Δ*yutF	Derivative of 168; contains the 351-bp in-frame deletion in *yutF* (*ΔyutF*)	pNZT1-*Δ*yutF→168
BsΔP	Derivative of 168; contains 33-bp deletion of the *yutD* upstream region (ΔP)	pNZT1-ΔP→168
BsMTNyutF	Derivative of 168; contains transcriptional fusions of *yutF* upstream region to *lacZ* and P_*spac*_ to *yutF*, Em^R^	pMUTIN2-yutF→168
BsΔPMTNyutF	The same as BsMTNyutF, but *yutF* to *lacZ* transcriptional fusion contains ΔP, Em^R^	pMUTIN2-yutF→BsΔP
BsMTNΔyutF	The same as BsMTNyutF, but contains Δ*yutF*, Em^R^	pMUTIN2-yutF→BsΔyutF
BsA1 to 3 BsB1 to 3	Derivative of 168; contains transcriptional fusions of the respective *yutDEF* fragments (A1-A3, B1-B3) to the promoterless *lacZ* inserted into the *amyE* locus, Cm^R^	pA1 to 3→168 pB1 to 3→168
Plasmids		
pNZT1	Thermosensitive integration vector, Em^R^	[17]
pNZT1-ΔyutF	pNZT1 derivative to introduce Δ*yutF*	The DNA fragment of *B*. *subtilis* 168 was amplified using OE-PCR (primers (+)yutFs_SalI, (-)yutFs_del, (+)yutFs_del, and (+)yutFs_PstI), *Sal*I-*Pst*I digested and cloned into *Sal*I-*Pst*I-digested pNZT1
pNZT1-ΔP	pNZT1 derivative to introduce ΔP	The DNA fragment of *B*. *subtilis* 168 was amplified using OE-PCR (primers (+)yutFs_Xho, (-)yutFs_Pdel, (+)yutFs_Pdel, and (-)yutFs_HindIII), *Xho*I-*Hin*dIII digested and cloned into *Xho*I-*Hin*dIII-digested pNZT1
pMWAL1-Prep	pBS72-based low copy shuttle expression vector containing the *repAB* promoter (P_*rep*_) of pLF1311 [19]; Ap^R^ (*E*. *coli*), Cm^R^ (*B*. *subtilis*)	[20]
pMWAL1-Prep-yutF	pMWAL1-Prep derivative for *yutF* expression controlled by P_*rep*_	The DNA fragment of *B*. *subtilis* 168 containing the SD sequence and coding sequence of *yutF* was PCR-amplified (primer pair (+)yutFs_XbaI/(-)yutFs_SmaI), *Xba*I-*Sma*I digested and cloned into *Xba*I-*Sma*I-digested pMWAL1-Prep
pET-15b	*E*. *coli* expression vector, Ap^R^	Novagen
pET15-H6-yutF	pET-15b derivative for the production of YutF with an N-terminal hexahistidine tag	Coding sequence of the *B*. *subtilis yutF* was PCR-amplified (primer pair (+)yutFs_NcoI/(-)yutFs_XhoI), digested with *Nco*I*-Xho*I and cloned into *Nco*I*-Xho*I-digested pET-15b
pMUTIN2	pBR322-based integration vector for *B*. *subtilis*; contains a multiple cloning site downstream of the IPTG-inducible P_*spac*_ promoter and a promoter-less *lacZ* gene; Ap^R^ (*E*. *coli*), Em^R^ (*B*. *subtilis*)	[21]
pMUTIN2-yutF	pMUTIN2 derivative; contains a fragment of the *B*. *subtilis yutF* gene. Used for simultaneous integration of the *lacZ* transcriptional reporter for monitoring *yutF* expression and the P_*spac*_ promoter for inducible expression of *yutF*	The *B*. *subtilis yutF* region (nt -55 to +283 with respect to the *yutF* translation start) was PCR-amplified (primer pair BsC/Bs3), digested with *Eco*RI-*Bam*HI and cloned into *Eco*RI-*Bam*HI-digested pMUTIN2
pDG268	Vector for integration of transcriptional *lacZ* reporter fusions into the chromosomal *amyE* locus of *B*. *subtilis* via a double-crossover event, Ap^R^ (*E*. *coli*), Cm^R^ (*B*. *subtilis*)	[22]
pA1-3, pB1-3	pDG268 derivatives; contains various fragments of the *yutDEF* region (see below) for monitoring *lacZ* reporter expression	The *B*. *subtilis* DNA fragments were PCR-amplified (see below for the respective primer pairs), digested with *Eco*RI-*Bam*HI and cloned into *Eco*RI-*Bam*HI-digested pDG268
pA1	nt -1070 to -503 (with respect to the *yutF* translation start)	BsA/Bs1
pA2	nt -1070 to +56	BsA/Bs2
pA3	nt -1070 to +281	BsA/Bs3
pB1	nt -778 to -503	BsB/Bs1
pB2	nt -778 to +56	BsB/Bs2
pB3	nt -778 to +281	BsB/Bs3

^a^ Ap^R^, ampicillin resistance; Em^R^, erythromycin resistance; Cm^R^, chloramphenicol resistance; IPTG, β-D-l-thiogalactopyranoside

^b^ This work unless otherwise specified; VKPM, The Russian National Collection of Industrial Microorganisms; pNZT1-ΔyutF→168 denotes the strain constructed from *B*. *subtilis* 168 using the pNZT1-ΔyutF plasmid; OE-PCR, overlap extension polymerase chain reaction; PCR, polymerase chain reaction

**Table 2:** Primers used in this study.

Name	Sequence (5' to 3'), restriction sites are underlined	Application
(+)yutFs_SalI	tatgtcgacggaacgatgtacaatgg	pNZT1-ΔyutF
(-)yutFs_del	cagtcagagagtcaatggcgtcgtatgtaatggaacggtcg	pNZT1-ΔyutF
(+)yutFs_del	cgaccgttccattacatacgacgccattgactctctgactg	pNZT1-ΔyutF
(+)yutFs_PstI	ttctgcagaaaaaatatcatccg	pNZT1-ΔyutF
(+)yutFs_XhoI	aaactcgagatcatctggcgtttttgtcattc	pNZT1-ΔP
(-)yutFs_Pdel	cctcttttttcactcattccctaaagaataaatcatgcgatttggc	pNZT1-ΔP
(+)yutFs_Pdel	tcgcatgatttattctttagggaatgagtgaaaaaagaggtgagatc	pNZT1-ΔP
(-)yutFs_HindIII	aaaaaagcttcgattaacagatggcctatacgc	pNZT1-ΔP
(+)yutFs_XbaI	aatctagaactggaaagccacaggagtg	pMWAL1-P_rep_-yutF
(-)yutFs_SmaI	ttcccgggtacctttttagatgacatcgtcg	pMWAL1-P_rep_-yutF
(+)yutFs_NcoI	ttaccatgggcagcagccatcatcatcatcatcacagcagcggcaaaacatataaagggtatttaattgatttagacg	pET15-H6-yutF
(-)yutFs_XhoI	gatcctcgagtcaaatgtatggaatccattcagtc	pET15-H6-yutF
BsA	ttgaattcttttgatagcggacatagcc	pA1, pA2, pA3
BsB	aagaattcaaaaagaggtgagatcatgattc	pB1, pB2, pB3
BsC	cgcgaattctaggacctgtttccgcgtt	pMUTIN2-yutF
Bs1	taggatccttcaagacaaaatacgcacagc	pA1, pB1
Bs2	taggatccgtgccattgtacatc	pA2, pB2
Bs3	cgcggatcctccccaatcacatacaca	pMUTIN2-yutF, pA3, pB3

### Growth conditions and crude cell extract preparation

*E*. *coli* and *B*. *subtilis* were grown in Luria-Bertani (LB) or M9 minimal medium [23] supplemented with D-glucose (0.4% for *E*. *coli* or 2% for *Bacillus*). When required, thiamine HCl (5 μg/ml), amino acids (40 μg/ml), casamino acids (0.1% (w⁄v)), ampicillin (100 μg/ml), erythromycin (200 μg/ml for *E*. *coli* or 10 μg/ml for *Bacillus*) or chloramphenicol (7 μg/ml) were added to the medium. Solid medium was obtained by adding 20 g/l agar to the liquid medium. If necessary, IPTG was added to the medium to a final concentration of 0.1 or 1 mM. All reagents were purchased from Sigma-Aldrich (Germany) unless otherwise specified.

Crude cell extracts to examine phosphatase activity were prepared by sonicating the cells grown with aeration to mid-log phase in M9 supplemented with glucose, tryptophan and casamino acids. β-galactosidase activity was measured in cultures grown with aeration to the mid-log phase in M9 supplemented with glucose, tryptophan and casamino acids or in phosphate-free minimal medium (100 mM Tris-Cl (pH 7.0), 1 g/l NH_4_Cl, 0.5 g/l NaCl, 0.5 g/l KCl, 2 mM MgSO_4_, 0.1 mM CaCl_2_) supplemented with glucose and tryptophan. If indicated, KH_2_PO_4_ (1 mM) was added to the phosphate-free minimal medium as a phosphate source.

### Genetic methods and DNA manipulation

All recombinant DNA manipulations were conducted according to standard procedures [24] and the recommendations of the enzyme manufacturer (Thermo Scientific). Plasmid and chromosomal DNA were isolated using the Qiagen Miniprep kit (Qiagen) and the Qiagen DNA purification kit (Qiagen), respectively, according to the manufacturer’s instructions.

Transformation of *B*. *subtilis* competent cells, PCR amplifications and DNA sequence analyses were performed as previously described [17]. Primers were purchased from Evrogen (Moscow, Russia). All constructions involving a PCR step were verified by DNA sequencing. Chromosomal deletion of *yutF* was confirmed by PCR (S1 Fig) and DNA sequencing.

### Heterologous YutF expression and purification

The expression construct, pET15-H6-yutF, was transferred into *E*. *coli* BL21(DE3). The recombinant protein Ht-YutF was overexpressed in the obtained transformants as previously described [25] and purified by immobilized-metal affinity chromatography on a HisTrap HP column (GE Healthcare) according to the manufacturer’s instructions. Imidazole-eluted recombinant protein was transferred to buffer A (50 mM HEPES, 10 mM MgCl_2_, 2 mM DTT, pH 7.4, 20% [v/v] glycerol) by gel filtration on a Sephadex G-25 column (Pharmacia) and stored at –70°C until analysis. The protein concentration was assayed using the Bio-Rad protein assay kit (Bio-Rad) with bovine serum albumin as a standard. The production, subunit size and protein purity were determined using 15% sodium dodecyl sulfate-polyacrylamide gel electrophoresis (SDS-PAGE). The proteins were stained with Coomassie Brilliant Blue R-250. Broad-range molecular weight markers (Unstained Protein Molecular Weight Marker, Thermo Scientific) were used as reference proteins.

Gel filtration analysis was performed on a Superdex 200 HR 10/30 column (Amersham Biosciences) in 50 mM potassium phosphate buffer at pH 7.5 containing 5 mM MgCl_2_ and 0.3 M NaCl at 4°C. The column was calibrated using a sample from a molecular-mass standard kit (Sigma*-*Aldrich).

### Enzymatic assays

General phosphodiesterase activity was measured spectrophotometrically at 25°C in a reaction mixture (1 ml) containing 50 mM Tricine buffer (pH 8.5), 0.5–5 mM Me^2+^ (Mg^2+^ or Mn^2+^), 5 mM Bis(*p*-nitrophenyl) phosphate (bis-*p*NPP) or 5 mM *p-*nitrophenyl phosphorylcholine (*p*NPPC) as a substrate and the purified Ht-YutF (0.12 μg). The reaction was started by substrate addition and *p*-nitrophenol (*p*NP) production was monitored at 410 nm (ε_410 nm_ = 15,460 M^-1^cm^-1^) [26].

General phosphatase activity toward the artificial substrate *p*NPP (*p*NPPase) was assayed spectrophotometrically at 25°C. The standard reaction mixture (1 ml) contained 100 mM Tris-HCl buffer, pH 8.9, 5 mM MgCl_2_, 5 mM *p*NPP and the purified Ht-YutF (0.12 μg) or crude cell extract (5 mg of total protein). The reaction was started by the addition of *p*NPP and monitored by continuously following the production of *p*NP at 410 nm [27]. No activity was detected in the control reaction, which excluded the enzyme.

Phosphatase (nucleotidase) activity toward the physiological substrates 5'-XMP, 5'-IMP, dIMP, 5'-IDP, 5'-ITP, 5'-GMP, dGMP, 5'-GDP, 5'-GTP, 5'-AMP, dAMP, 5'-ADP, 5'-ATP, 3'-AMP, 5'-CTP, 3'-CMP, 5'-UMP, 5'-UTP, UDP-glucose, Glucose-6P (G6P), PRPP or R5P was assayed by the rate of released inorganic phosphate (Pi). The standard reaction mixture (0.25 ml) contained 100 mM 4-morpholineethanesulfonic acid (MES) buffer, pH 6.0, 5 mM MgCl_2_, 5 mM of substrate and the purified Ht-YutF (0.12 μg). The assay was started by substrate addition and was carried out at 37°C for 25 min. The reaction rate was linear under these conditions. The amount of released Pi was assessed colorimetrically [28], and the concentration was estimated from a standard curve obtained with KH_2_PO_4_. To exclude the influence of non-enzymatic factors, the background phosphate level was monitored in parallel using a control reaction without the enzyme. The activity was calculated by subtracting the nonspecific substrate hydrolysis measured in the absence of Ht-YutF, which was no more than 5% of activity.

The pH dependence of the phosphatase activity toward *p*NPP (5 mM) or 5'-IMP (5 mM) was determined in the presence of 5 mM MgCl_2_ and purified Ht-YutF. The assays were performed in the following buffer systems (100 mM): MES buffer between pH 5.5 and 6.5, imidazole buffer between pH 6.0 and 7.5, Tris-HCl buffer between pH 7.1 and 8.9, and CHES buffer between pH 8.6 and 10.0.

The metal dependence of the phosphatase activity of the purified Ht-YutF toward *p*NPP (5 mM) was determined at pH 8.9 using various divalent metal ions (Mg^2+^, Mn^2+^, Co^2+^, Ca^2+^, Zn^2+^, Ni^2+^, Cu^2+^ and Ba^2+^) at two concentrations: 0.5 and 5 mM.

The kinetic parameters for Ht-YutF were determined using the appropriate activity assay with at least eight different substrate concentrations ranging from 0 to 5 mM for *p*NPP and PRPP, from 0 to 12 mM for R5P and 5'-XMP, and from 0 to 20 mM for 5'-GMP. The data were analyzed by nonlinear regression using GraphPad Prism 6 software (GraphPad Software Inc., San Diego, CA, USA). The *k*_*cat*_ values were calculated based on the subunit molecular mass of Ht-YutF. All kinetic parameters were obtained from at least three measurements. The values for phosphatase activities toward *p*NPP or natural substrates are presented as the amount (nanomoles) of *p*NP or Pi, respectively, released per min under standard conditions.

The β-galactosidase activity assay was performed as described by Miller [23]. The β-galactosidase activity values are presented as Miller units (MU).

### Statistical analysis

Statistical analyses were performed using GraphPad Prism 6 software.

## Results and Discussion

### Search for genes encoding 5'-nucleotidases in *B*. *subtilis*

**Fig 1:**
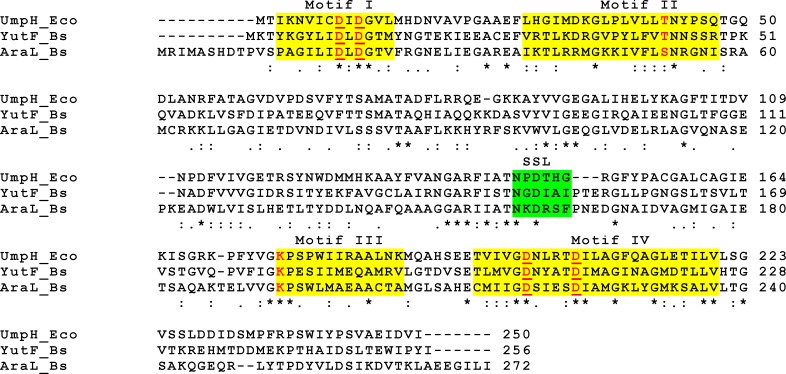
Comparison of the deduced amino acid sequence of *B*. *subtilis* YutF with characterized members of a type IIA subfamily of HADSF. The conserved residues involved in catalysis are shown in red, and the residues required for coordinating the Mg ion in the active site are underlined. Approximate areas of the four conserved motifs (I-IV) are shaded in yellow. The conserved residues from the cap domain C2 that can act as a substrate specificity loop (SSL) are shaded in green. Similar (‘.’ and ‘:’) and identical (‘*’) amino acids are indicated. The following protein sequences were used (GenBank accession numbers are indicated in parentheses): YutF_Bs, putative hydrolase from *B*. *subtilis* (NP_391109.1); UmpH_Eco, UMP phosphatase from *E*. *coli* (NP_415201.1); and AraL_Bs, sugar phosphatase from *B*. *subtilis* (NP_390755.1). The multiple sequence alignment was generated using the CLUSTALW program [34].

Despite the important role of 5'-nucleotidases in cellular metabolism and the completely sequenced genome of *B*. *subtilis*, only a few genes encoding these enzymes have been characterized in this Gram-positive bacterium, a workhorse industrial microorganism included in the Food and Drug Administration’s GRAS (generally regarded as safe) list. A search for genes orthologous to characterized genes with a certain function is a suitable tool to identify genes with the same function in other genomes. Orthologs are genes in different species that evolved from a common ancestral gene by speciation, whereas paralogs are genes related by duplication within a genome [29]. Based on this evolutionary relationship, orthologs are generally assumed to have equivalent functions across different organisms, while paralogs are considered a source of functional innovation. Therefore, discrimination between orthologs and paralogs is critical for the reliable prediction of gene functions. One commonly used simple method to find orthologs is a bidirectional search in two genomes to identify reciprocal best hits (RBHs). RBHs are proteins encoded by two genes, each in a different genome, that find each other as the best scoring match in the other genome [29]. To identify genes encoding *B*. *subtilis* 5'-nucleotidases using this approach, a homology search with the amino acid sequence of 5'-nucleotidase UmpH from *E*. *coli* as a query against the *B*. *subtilis* genome was performed using the BLASTp algorithm introduced by NCBI [30]. The deduced amino acid sequence of *B*. *subtilis yutF* was the best hit (E-value of 9x10^-43^). YutF demonstrated high amino acid sequence similarity to UmpH (31% identity and 54% similarity) (Fig 1). In this search, the *B*. *subtilis* sugar phosphatase AraL was also found as a UmpH homolog; however, the E-value was higher than that for YutF (1x10^-15^ vs. 9x10^-43^). AraL displayed 26% (46%) amino acid identity (similarity) to UmpH and 29% (50%) amino acid identity (similarity) to YutF (Fig 1). The new BLASTp search using the amino acid sequence of YutF as the query against the *E*. *coli* genome yielded UmpH as the best hit (E-value of 9x10^-43^). This RBH search result indicated that YutF is the most likely candidate for an UmpH ortholog in *B*. *subtilis*. YutF consists of 256 amino acids and has a monomer molecular mass of 28 kDa. An analysis of the protein sequence using the signal peptide prediction software Signal P 4.1 [31] and the topology prediction programs SOSUI 1.11 and TMHMM 2.0 [32,33] revealed no evidence for the presence of an N-terminal signal peptide or transmembrane helices, suggesting an intracellular localization of YutF.

UmpH, AraL and YutF are members of the large subfamily IIA of the HADSF (http://www.ebi.ac.uk/interpro/entry/IPR006357). All members of this subfamily contain a highly conserved α/β core domain that supports a catalytic scaffold, and a variable cap domain that desolvates the active site for catalysis and confers substrate specificity [14]. The active site of the core domain is formed by four loops that correspond to sequence motifs I-IV (Fig 1**)**. The cap domain C2 is situated between the second and third motif (UmpH residues 71–175) and comprises the amino acid residues involved in substrate recognition, which is often called the substrate specificity loop (UmpH residues 144–149) [10].

The enzymatic activities and physiological roles of the majority of the IIA subfamily representatives have not yet been identified. Based on the presence of conserved domains, YutF has been annotated in the NCBI Protein database (http://www.ncbi.nlm.nih.gov/protein) as an uncharacterized hydrolase or putative *p*-nitrophenyl phosphatase.

**Fig 2:**
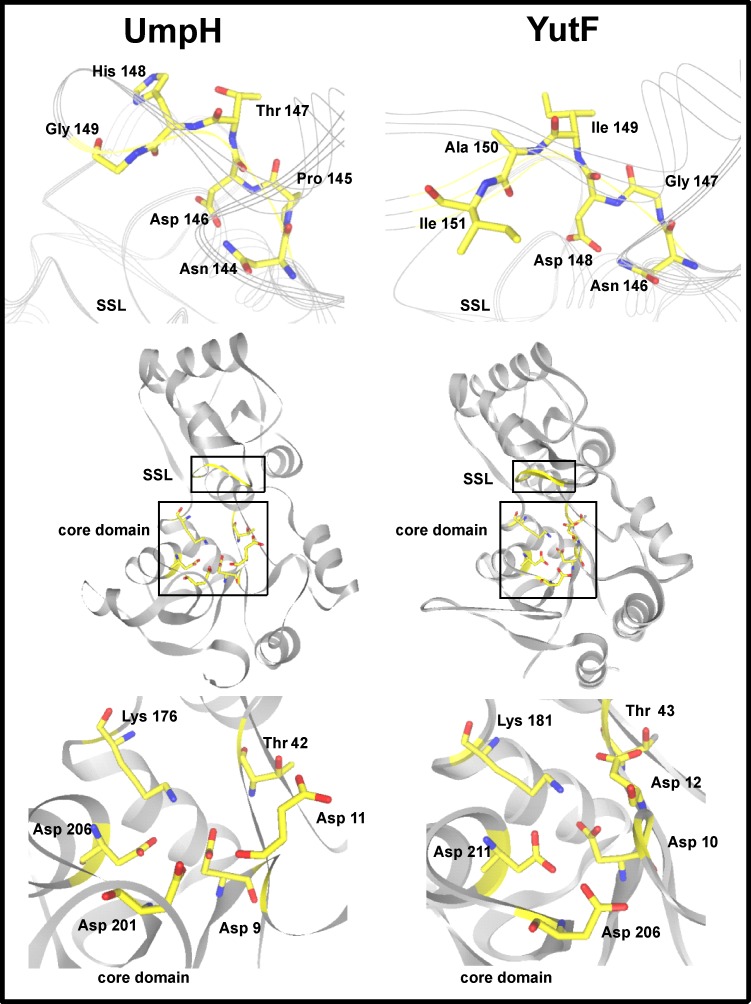
Comparison of the 3D structures of UmpH and YutF. Ribbon diagram representations of the 3D structures of UmpH (PDB id: 2c4n) and YutF (PDB id: 3pdw) (in the center) and magnified views of the substrate specificity loop (SSL) and the core domain configurations (on the top and bottom, respectively). The core domain and SSL residues are shown in yellow, and their regions are highlighted by black boxes. The identities of conserved residues involved in catalysis are indicated. This figure was prepared using 3D*-*Mol Viewer (a component of Vector NTI Advance 10 software, https://www.invitrogen.com/).

A comparison of the crystal structures of UmpH (PDB id: 2c4n) [10] and the solved, but unpublished, YutF (PDB id: 3pdw) showed that both proteins share similar catalytic residues at the active site (Asp9, Asp11, Thr42, Lys176, Asp201 and Asp206 in UmpH vs. Asp10, Asp12, Thr43, Lys181, Asp206 and Asp211 in YutF) (Fig 2). The structural similarity and identity of the conserved catalytic residues of the core domain suggest that YutF and UmpH may be functional homologs. However, the sequence motif of UmpH, NPDTHG, which forms the substrate specificity loop, coincides with a corresponding YutF sequence at only two of six positions, suggesting that UmpH and YutF possess different substrate spectra (Figs 1 and 2).

To determine whether the *yutF*-encoded protein could function as a *p*NPPase, strains with different levels of *yutF* expression were constructed based on *B*. *subtilis* 168. To eliminate YutF activity, *yutF* was disrupted in the chromosome of strain 168, yielding the strain BsΔyutF. To provide plasmid-borne expression of *yutF* from the “strong” constitutive promoter *repAB* (P_*rep*_), the low-copy plasmid pMWAL1-Prep-yutF was constructed and introduced into BsΔyutF, yielding the strain BsΔyutF (pMWAL1-Prep-yutF). The phosphohydrolase activity against the general phosphatase substrate, *p*NPP, was tested in crude extracts of the resulting strains (Table 3). The inactivation of *yutF* had essentially no effect on cell growth in rich or minimal medium (data not shown) but resulted in a drop in *p*NPP hydrolysis in the crude extracts of *B*. *subtilis* cells to undetectable levels (Table 3). In the Δ*yutF* background, *yutF* expression from P_*rep*_ led to a significant enhancement of phosphohydrolase activity with respect to *p*NPP (Table 3). These data suggested that the product of *yutF* is responsible for the major *p*NPPase activity in *B*. *subtilis* cells. To further investigate the biochemical function of YutF, the recombinant protein was expressed in *E*. *coli*, purified and characterized.

**Table 3:** *p*NPPase activity in strains with various levels of *yutF* expression.

Strain	Specific *p*NPPase activity, nmol min^-1^ mg^-1^
*B*. *subtilis* 168	24.5 ± 2.5
BsΔyutF	<1
BsΔyutF (pMWAL1-Prep-yutF)	542.2 ± 58.0
BsΔP	6.7 ± 0.9
*B. subtilis* 168 (pMWAL1-Prep-yutF)	1060.0 ± 90.0
BsΔP (pMWAL1-Prep-yutF)	473.2 ± 45.0

The *p*NPPase activity was assayed spectrophotometrically in a standard reaction mixture containing 100 mM Tris-HCl buffer, pH 8.9, 5 mM MgCl_2_, 5 mM *p*NPP and 5 mg of crude cell extract total protein (see Materials and methods). The specific activity is presented as nanomoles of *p*NP released per min per milligram of total protein. The results are expressed as the means ± standard errors of at least three independent experiments.

### Heterologous expression and purification of YutF

The N-terminal hexahistidine-tagged YutF protein was produced in soluble form in the *E*. *coli* strain BL21(DE3) from the expression construct pET15-H6-yutF. The electrophoretic patterns of total extracted proteins by sodium dodecyl sulfate-polyacrylamide gel electrophoresis (SDS-PAGE) revealed a protein band with a molecular mass of approximately 30 kDa, which was consistent with predicted molecular mass of YutF containing the N-terminal hexahistidine tag (29.2 kDa). Moreover, this band was not detected in the control strain (S2 Fig). The heterologously produced enzyme was purified to homogeneity from the supernatant of the disrupted cells using immobilized-metal affinity chromatography. The typical yield of the purified recombinant His-tagged protein YutF (Ht-YutF) was approximately 4.5 mg from 250 ml of culture, and the purity of Ht-YutF was greater than 95% (S2 Fig). The subunit structure of Ht-YutF was analyzed by gel filtration. The protein eluted as a single symmetric peak with a retention time that corresponded to a molecular mass of approximately 65 ± 10 kDa, which is about twice the predicted mass of the monomer, indicating that Ht-YutF likely exists as a dimer in solution. In agreement with this result, an analysis of probable assemblies in the crystal using the PDBePISA server (Protein Interfaces, Surfaces, and Assemblies service PISA at the European Bioinformatics Institute, http://www.ebi.ac.uk/msd-srv/prot_int/pistart.html) showed that YutF likely exists as a stable dimer in solution. The dimer is stabilized by thirteen hydrogen bonds and ten salt bridges (distances < 3.8 Å) and has an interface with a surface area per monomer of ∼1202 Å^2^, which is approximately 10% of the total surface area of a single monomer (∼12044 Å^2^).

### Biochemical characterization of recombinant Ht-YutF

An analysis of the three-dimensional structure of YutF demonstrated that the N-terminal amino acid residues are not involved in the formation of an active site or in dimer formation. Therefore, we predicted that the histidine tag at the N-terminus of YutF would not alter the catalytic properties of the enzyme. In agreement with this result, recombinant Ht-YutF was shown to possess phosphohydrolase activity toward *p*NPP (Table 4).

**Table 4:** Study of the substrate specificity of recombinant Ht-YutF.

Substrate	A (nmol min^-1^ mg^-1^)
*p*NPP	89000 ± 8000
R5P	690 ± 60
5'-XMP	510 ± 40
PRPP	260 ± 30
5'-IMP	79 ± 7
5'-GMP	74 ± 7
dGMP	71 ± 6
dIMP	62 ± 6
5'-UMP	42 ± 4
5'-AMP	28 ± 3
5'-IDP	26 ± 3
5'-CTP	25 ± 3
5'-UTP	15 ± 2
5'-CMP	14 ± 2
dAMP	13 ± 2
5'-ITP	12 ± 2
5'-GDP	11 ± 2
G6P	9 ± 1
5'-GTP	6 ± 1
5'-ADP	4 ± 1
5'-ATP	4 ± 1

The rates of hydrolysis of *p*NPP or physiological substrates by purified Ht-YutF (0.12 μg) were measured by continuously following the production of *p*NP (at 410 nm) or Pi, respectively, under standard conditions as described in Materials and methods. The specific activity is presented as nanomoles of *p*NP or Pi released per min per milligram of protein. The results are expressed as the means ± standard errors of at least three independent experiments. No activity was detected using the other tested substrates (3'-CMP, 3'-AMP, and UDP-glucose).

During the general phosphatase screening, Ht-YutF demonstrated no activity toward bis-*p*NPP or *p*NPPC, suggesting an absence of phosphodiesterase activity (data not shown).

The metal dependence of the Ht-YutF activity toward *p*NPP was determined using various divalent metal ions (S3 Fig). Similarly to UmpH and other characterized HADSF proteins belonging to the type IIA subfamily [11,16,35], Ht-YutF has an absolute requirement for Mg^2+^ for its activity. It was estimated that the optimal concentration of Mg^2+^ was 5 mM.

The optimum pH for Ht-YutF was estimated to be between 8.7 and 9.0 in 100 mM Tris-HCl buffer with *p*NPP as a substrate and between 6.0 and 6.5 in 100 mM MES buffer with 5'-IMP as a substrate (S4 Fig).

Based on these findings, the phosphatase activity of purified Ht-YutF with respect to physiological substrates such as deoxyribo- and ribonucleoside tri-, di- and monophosphates, sugar phosphates and other phosphorylated metabolites was evaluated under standard conditions as described in the Materials and methods section.

Ht-YutF demonstrated a relatively high phosphohydrolase activity toward R5P, 5'-XMP and PRPP and possessed a modest activity toward various nucleotides, hydrolyzing predominantly 5'-nucleoside monophosphates (Table 4). The enzyme exhibited a higher specific activity toward 6-oxopurine-containing ribo- and deoxyribonucleoside monophosphates (5'-XMP, 5'-IMP, 5'-GMP, dGMP and dIMP) than toward 6-aminopurine-containing AMP and dAMP and pyrimidine nucleoside monophosphates. Ht-YutF showed no detectable reactivity with ribonucleoside 3'-monophosphates. In contrast to UmpH, which hydrolyzed ribonucleoside phosphates but not deoxyribonucleoside phosphates, Ht-YutF showed poor discrimination between ribo- and deoxyribonucleoside monophosphates and a better ability to distinguish between purine/pyrimidine moieties. The enzyme did not show appreciable activity against G6P or UDP-glucose.

The kinetic parameters of Ht-YutF toward the most preferable substrates were studied (Table 5, S5 Fig). The experimental data fit well to hyperbolic curves and were described by Michaelis-Menten kinetics. The recombinant protein demonstrated rather low substrate specificity and catalytic efficiencies for the tested physiological substrates. However, its *K*_*m*_ values fell within the range of *K*_*m*_ values for other characterized nucleotidases represented in the BRENDA database (0.01–56 mM), and the catalytic efficiencies corresponded to those of the 5'-nucleotidase UmpH and of another member of the type IIA subfamily of HADSF from *B*. *subtilis*, the sugar phosphatase AraL [10,16]. The maximal initial velocity was observed for the general substrate *p*NPP, but the affinity of Ht-YutF for *p*NPP was nearly the same as that for 5'-XMP or PRPP. Interestingly, Ht-YutF demonstrated a high *K*_*m*_ for R5P that exceeded the range of known bacterial physiological concentrations (approximately 0.5 mM for *B*. *subtilis*), but the *k*_*cat*_ for this substrate was several times higher than the *k*_*cat*_ for 5'-XMP or PRPP. These characteristics of the enzyme might be required when the intracellular concentration of a substrate in cells (or its local concentration in certain cell compartments) reaches extremely high values and an immediate reduction of the respective pools via dephosphorylation is necessary.

**Table 5:** Kinetic parameters of recombinant Ht-YutF.

Substrate	*K*_*m*_ (mM)	*k*_*cat*_ (s^-1^)	*k*_*cat*_/*K*_*m*_ (s^-1^ M^-1^)
*p*NPP	1.64 ± 0.06	61.0 ± 1.0	37000
5'-XMP	1.53 ± 0.11	0.31 ± 0.01	210
PRPP	1.27 ± 0.10	0.17 ± 0.01	130
R5P	24 ± 2	1.84 ± 0.12	77
5'-GMP	6.65 ± 1.26	0.06 ± 0.01	9.0

The kinetic parameters were determined using the respective activity assay with at least eight different substrate concentrations as described in Materials and methods. The results are expressed as the means ± standard errors of at least three independent experiments.

### *yutD*-*yutE*-*yutF* form a three-cystronic operon with increasing expression in response to YutF overproduction

**Fig 3:**
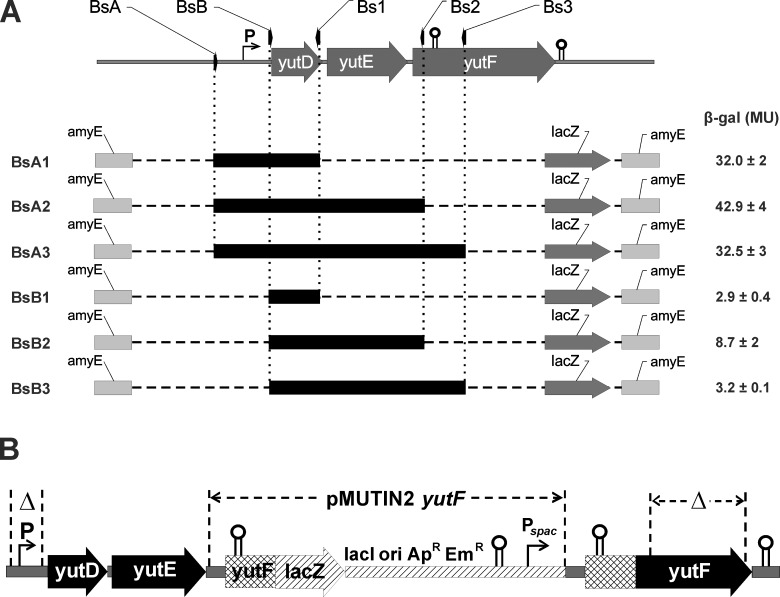
Schematic representation of the *B*. *subtilis* 168 *yutDEF* region in the constructed strains. (A). Left: The *B*. *subtilis* 168 *yutD*-*yutE*-*yutF* region (top) and chromosomal transcription fusions of the *yutF* region to a promoterless *lacZ* in derivatives of *B*. *subtilis* 168, strains BsA1, BsA2, BsA3, BsB1, BsB2 and BsB3 (bottom). The *yutD*-*yutE*-*yutF* region fragments fused to a promoterless *lacZ* are denoted by thick black lines. Promoters (P and P_*spac*_) and rho-independent transcription terminators are indicated. Right: specific β-galactosidase activity (Miller units, MU) of crude cell extracts from the indicated strains. The values are the means ± standard errors of at least three independent experiments. (B). The *yutDEF* region in pMUTIN2-yutF-containing strains. The deleted fragments in the *yutDEF* promoter region and in the *yutF* coding region in BsΔPMTNyutF and BsMTNΔyutF, respectively, are indicated by Δ.

*B*. *subtilis yutF* is located 29 bp downstream of *yutE*, which in turn is located 24 bp downstream of *yutD* (Fig 3A). No potential promoters were observed in the upstream regions of the *yutE* and *yutF* ORFs, whereas the 5' TTGATG-N17-TATGAT 3' sequence, which shares similarities with consensus sequences from known SigA-promoters, was found upstream of the translational start codon of the *yutD* ORF. This *in silico* analysis is in agreement with transcriptome analysis data for this chromosome region in *B*. *subtilis*, demonstrating the presence of a single transcriptional unit comprising *yutD*, *yutE* and *yutF* ORFs [36]. Two putative Rho*-*independent transcription terminators were predicted in the *yutD-yutE-yutF* region using ARNold Finding Terminators software (http://rna.igmors.u-psud.fr/toolbox/arnold/index.php) [37]. The first terminator is located at the 5' end of the coding region of *yutF* (tgaatttgtcagaacgctgaaagatcgcggcgttccttatcttttcgt, ΔG = -11.5 kcal/mol), and the second one is located downstream of the *yutF* stop codon (acatttgaaaaaagggcgccctaaaagggtgcccttattctgtatgccgc, ΔG = -13.9 kcal/mol). These data suggested that *yutD*, *yutE* and *yutF* constitute an operon and that *yutF* expression might be subjected to additional regulation through the premature termination of transcription.

To evaluate whether transcription of the *yutF* gene was initiated from a presumed promoter upstream of the *yutD* gene (designated as P), the chromosomal region (from -60 bp to -28 bp with respect to the *yutD* translation start site, hereafter referred to ΔP) (Fig 3B) was deleted in the chromosome of *B*. *subtilis* 168 to yield the strain BsΔP. The significantly lower phosphohydrolase activity toward *p*NPP in crude cell extracts of BsΔP than *B*. *subtilis* 168 suggested a dominant role of this sequence in *yutF* gene expression (Table 3).

To further investigate *yutF* expression, a series of single-copy transcriptional fusions containing different fragments of the *yutD*-*yutE*-*yutF* region fused to a promoterless *lacZ* reporter gene were constructed (Fig 3A). Each fusion construct was integrated at the *amyE* locus of the *B*. *subtilis* 168 chromosome to yield strains BsA1, BsA2, BsA3, BsB1, BsB2 and BsB3. The β-galactosidase activity was tested in these strains (Fig 3A). In BsA1, BsA2 and BsA3, the DNA fragment fused to *lacZ* (corresponded to a region starting 308 bp upstream of *yutD* and extending into *yutD* or *yutF*) included the presumed promoter P (Fig 3A). These strains demonstrated fairly high β-galactosidase activities. A 292 bp reduction in the size of the fragments in the fusion construct at the 5' end resulted in a drastic drop in β-galactosidase activity, which was most likely due to the loss of the promoter P sequence (strains BsB1, BsB2 and BsB3, Fig 3A). These data confirmed that *yutD*, *yutE* and *yutF* formed a three-cystronic operon, which was transcribed from the promoter located upstream of *yutD*. However, some residual β-galactosidase activity was observed in strains BsB1, BsB2 and BsB3. These data correlated with the residual level of *p*NPPase activity in BsΔP (Table 3) and might indicate the presence of an additional promoter(-s) between the promoter P sequence and *yutF*, ensuring a low level of expression at least under the present experimental conditions. The transcriptional *lacZ* fusions in BsA2 and BsA3 and in BsB2 and BsB3 differ from each other by the presence of a Rho-independent transcription terminator-like sequence located at the beginning of the N-terminal coding sequence of *yutF* (Fig 3A). The strains BsA2 and BsB2 demonstrated higher β-galactosidase activity than BsA3 and BsB3, respectively, suggesting the involvement of the stem-loop structure in premature transcription termination.

The expression of *yutF* was further examined in strains BsMTNyutF, BsΔPMTNyutF and BsMTNΔyutF, which contain single-copy transcriptional fusions that were inserted directly into the *yutF* locus. A single cross-over event was used to place the pMUTIN2-yutF-borne promoterless *lacZ* reporter gene under the transcriptional control of the *yutF* upstream region, and the intact coding region of *yutF* under the control of the IPTG-inducible *spac* promoter, P_*spac*_, to yield the BsMTNyutF strain (Fig 3B). Additionally the strain BsΔPMTNyutF carried a 33-bp deletion of the promoter P sequence (from -60 bp to -28 bp with respect to the *yutD* translation start site), whereas BsMTNΔyutF contained a 351-bp in-frame deletion in the coding region of *yutF* that resulted in YutF-deficiency. The *yutF* expression level and YutF production were estimated in these strains using β-galactosidase and *p*NPPase activity assays, respectively. Unexpectedly, in response to IPTG addition, the BsMTNyutF strain exhibited a significant enhancement of not only *p*NPPase activity but also β-galactosidase activity (Table 6). The increases in both activities were directly proportional to the amount of IPTG in the medium. Moreover, an in-frame deletion of the *yutF* coding region, which prevented YutF production, completely reversed the IPTG-mediated enhancement of *lacZ* reporter expression (strain BsMTNΔyutF). These data indicated that *yutF* expression was positively regulated by YutF at the level of transcription. Furthermore, a significant decrease in β-galactosidase activity due to the deletion of the presumed promoter P sequence located upstream of the *yutD* gene (strain BsΔPMTNyutF) indicated that *yutF* expression is controlled by this promoter and further confirmed that *yutF* is a part of the *yutDEF* operon. The loss of β-galactosidase activity induction by IPTG in BsΔPMTNyutF indicated that *yutF* expression and its positive regulation by YutF are both controlled by the same regulatory elements located upstream of *yutD*.

**Table 6:** The influence of YutF production on *yutF* expression in strains with pMUTIN2-borne transcriptional fusions.

Strain	IPTG, mM	β-galactosidase (MU)	Specific *p*NPPase, nmol min^-1^ mg^-1^
BsMTNyutF	-	41.3 ± 3.0	<1
BsMTNyutF	0.1	134.1 ± 2.4	57.5 ± 3.0
BsMTNyutF	1	302.5 ± 5.3	90.5 ± 1.5
BsΔPMTNyutF	-	7.2 ± 0.8	<1
BsΔPMTNyutF	1	8.1 ± 0.5	90.0 ± 1.0
BsMTNΔyutF	-	45.3 ± 0.6	<1
BsMTNΔyutF	1	41.3 ± 0.9	<1

β-galactosidase and *p*NPPase activities were measured as described in Materials and methods. IPTG was added to the growth medium to the indicated final concentrations. The results are expressed as the means ± standard errors of at least three independent experiments.

To further investigate the positive autoregulation of *yutF* expression, *B*. *subtilis* 168, BsΔP and BsΔyutF cells were transformed with the plasmid pMWAL1-Prep-yutF, and the resulting strains were evaluated for *p*NPPase activity (Table 3). The *p*NPPase activity levels in BsΔyutF (pMWAL1-Prep-yutF) and BsΔP (pMWAL1-Prep-yutF), which characterized the level of plasmid-borne expression of *yutF*, were almost equivalent (approximately 500 nmol min^-1^ mg^-1^); *p*NPPase activity in *B*. *subtilis* 168 (pMWAL1-Prep-yutF) was estimated to be 1060 nmol min^-1^ mg^-1^, as much as two times higher. This value significantly exceeded the algebraic sum of the *p*NPPase activities in strains *B*. *subtilis* 168 (the wild type *yutF*) and BsΔyutF (pMWAL1-Prep-yutF) (only plasmid-borne expression of *yutF*), confirming that YutF overproduction further activated its own expression.

**Fig 4:**
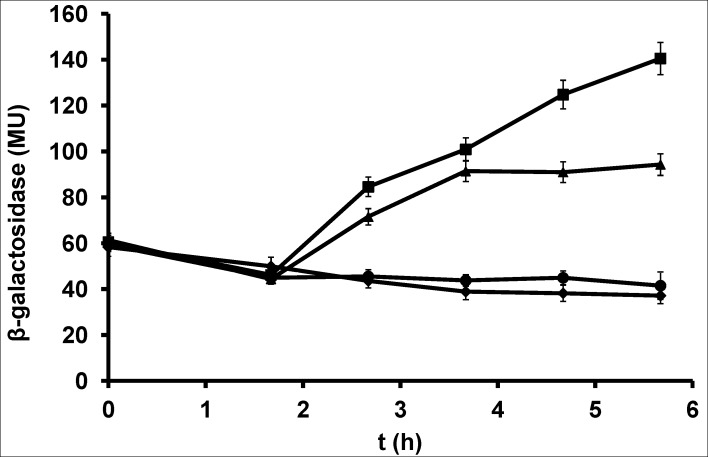
Effect of inorganic phosphate and IPTG on the induction of β-galactosidase in BsMTNyutF. β-galactosidase activity in BsMTNyutF during cultivation in glucose phosphate-free minimal medium without IPTG or KH_2_PO_4_ (circles), 1 mM IPTG without KH_2_PO_4_ (triangles), 1 mM KH_2_PO_4_ without IPTG (diamonds), and 1 mM IPTG and 1 mM KH_2_PO_4_ (squares) was measured as described in Materials and methods. The results are expressed as the means ± standard errors of at least three independent experiments.

Phosphate limitation induces genes encoding phosphate-liberating enzymes to provide sufficient inorganic phosphate for survival under phosphate starvation conditions [8]. However, *yutF* expression depends on the availability of phosphate in another manner. The β-galactosidase activity profiles in BsMTNyutF (reflecting the level of *yutF* expression) during growth in the presence of different levels of Pi and IPTG (for P_*spac*_-controlled induction of YutF expression) are presented in Fig 4. The specific β-galactosidase activities in cells growing in phosphate-free and phosphate-rich minimal medium were almost equivalent and were relatively low in the absence of YutF production (no IPTG in the medium). In the presence of IPTG, the specific β-galactosidase activities measured in BsMTNyutF growing under phosphate-limited conditions were lower than those observed in the presence of inorganic phosphate, suggesting that a positive effect of YutF on *yutDEF* expression is enhanced under phosphate-abundant conditions.

## Conclusions

Enzymes involved in the dephosphorylation of nucleotides, 5'-nucleotidases, are particularly important for maintaining the cellular balance of nucleotide and nicotinamide adenine dinucleotide pools. Thus, 5'-nucleotidases participate in the control of DNA replication, RNA synthesis and cellular energy. Soluble forms of 5'-nucleotidases belong to the HADSF family of proteins. One well-studied member of the HADSF subfamily IIA, the *E*. *coli* 5'-nucleotidase UmpH, has been shown to control the level of end products of the pyrimidine pathway [15]. With a significantly higher Michaelis constant (*K*_*m*_ of 0.12 mM) than the normal steady-state UMP concentration (0.052 mM), UmpH converts UMP to uridine only under conditions of UMP overproduction, thus decreasing intracellular UMP concentrations even in the presence of deregulated pyrimidine biosynthetic flux. *B*. *subtilis* has been shown to possess several intracellular enzymes with 5'-nucleotidase activity, but most of these respective genes have not been identified to date [38,39]. Recently, some HADSF members from *B*. *subtilis* were shown to catalyze the dephosphorylation of sugar phosphates, the riboflavin precursor and FMN, but these enzymes lacked activity toward the tested nucleotides [16,40]. To identify and characterize 5'-nucleotidases in *B*. *subtilis*, a BLAST search for UmpH homologs was performed in this bacterium. A putative hydrolase of the HADSF family encoded by the *yutF* gene was found to be the most likely candidate for an UmpH ortholog in *B*. *subtilis*. YutF was expressed in *E*. *coli* as an N-terminal hexahistidine-tagged protein and purified. Biochemical characterization of the recombinant YutF revealed that it is the major *p*-nitrophenyl phosphatase in *B*. *subtilis* and that it possesses phosphohydrolase activity toward multiple physiological substrates, including various 5'-nucleotides and their metabolic precursors. In contrast to UmpH, the most preferred natural substrates for the recombinant YutF are 5'-XMP, PRPP and R5P.

The UmpH-encoding gene, *nagD*, is a part of the divergent *nagE-nagBACD* operon, which is necessary for the utilization of N-acetylglucosamine as a carbon source in *E*. *coli* [41]. We found that the *yutF* gene is co-transcribed with the two upstream genes, *yutD* and *yutE*, which encode conserved hypothetical proteins that are not homologous to any characterized proteins. Therefore, the gene context of YutF within the *yutDEF* operon cannot help predict its physiological function in cellular metabolism. We showed that YutF overproduction increased the level of *yutDEF* operon expression, and this upregulation was enhanced in the presence of inorganic phosphate. HADSF phosphatases have a highly similar active site and catalyze the same fundamental chemistry as response regulator receiver domains of two-component signal transduction systems [42,43]. These systems allow organisms to sense and respond to changes in different environmental conditions [44]. Two-component signal transduction systems mostly consist of a membrane-bound histidine kinase that detects the signal and a response regulator that, in a phosphorylated form, executes the cellular response [45]. It is interesting that some of the response regulators consist of an isolated receiver domain (i.e., lacking an effector domain) and are able to regulate target effectors due to their own phosphorylation by small molecules (for example, acetyl phosphate) as phosphodonors [45,46,47]. We speculate that YutF can act in a similar way. When the intracellular pool of a certain phosphorylated compound, the YutF substrate, significantly increases, the protein interacts with this phosphodonor to form an intermediate phosphorylated form that is capable of activating the expression of the *yutDEF* operon. Our hypothesis was indirectly confirmed by recent studies that showed the ability of some the HADSF members to undergo conformational changes during catalysis [48,49]. Because no DNA-binding motif has been found in YutF, it probably exerts the control indirectly, altering the activity of an unknown regulator of *yutDEF* expression.

Genes homologous to *yutF* can be found in diverse Firmicutes, in which these genes are often associated with homologs of the open reading frames of *yutD* and *yutE*. To define the actual role of YutD, YutE and YutF in cellular physiology, further investigation is needed.

## Supporting Information

S1 Fig Confirmation of *yutF* deletion by PCR. Agarose (1%) gel electrophoresis of PCR products (4 μl) visualized by staining with ethidium bromide is shown. M, 1 kb DNA Ladder (Thermo Scientific). The figure shows colony PCR of *B*. *subtilis* 168 (Lane 1) and BsΔyutF (Lane 2). DNA was amplified using primers BsC and (+)yutFs_PstI.
(TIF)

S2 Fig Expression of recombinant Ht-YutF in *E*. *coli* and purification. Lanes: 1, cellular lysate of BL21(DE3) harboring pET15b(+) induced with IPTG (17 μg of total protein); 2, cellular lysate of BL21(DE3) harboring pET15-H6-YutF induced with IPTG (17 μg of total protein); 3, the purified Ht-YutF product (5 μg). M, molecular mass standard (Unstained Protein Molecular Weight Marker, Thermo Scientific). Protein samples were separated by SDS-PAGE and stained with Coomassie Brilliant Blue.
(TIF)

S3 Fig Divalent metal ion dependence of the phosphatase activity of purified Ht-YutF toward *p*NPP. (TIF)

S4 Fig pH dependence of the phosphatase activity of purified Ht-YutF toward (A) *p*NPP (5 mM) and (B) 5'-IMP (5 mM).
(TIF)

S5 Fig Substrate titration plots of Ht-YutF for (A) *p*NPP, (B) 5'-XMP, PRPP, R5P and 5'-GMP.
(TIF)
