# Supplementary figures and images for: Identification, Heterologous Expression, and Functional Characterization of Bacillus subtilis YutF, a HAD Superfamily 5'-Nucleotidase with Broad Substrate Specificity
Source: PLoS One. 2016 Dec 1;11(12):e0167580. doi: 10.1371/journal.pone.0167580 (PMC5132288; doi:10.1371/journal.pone.0167580)

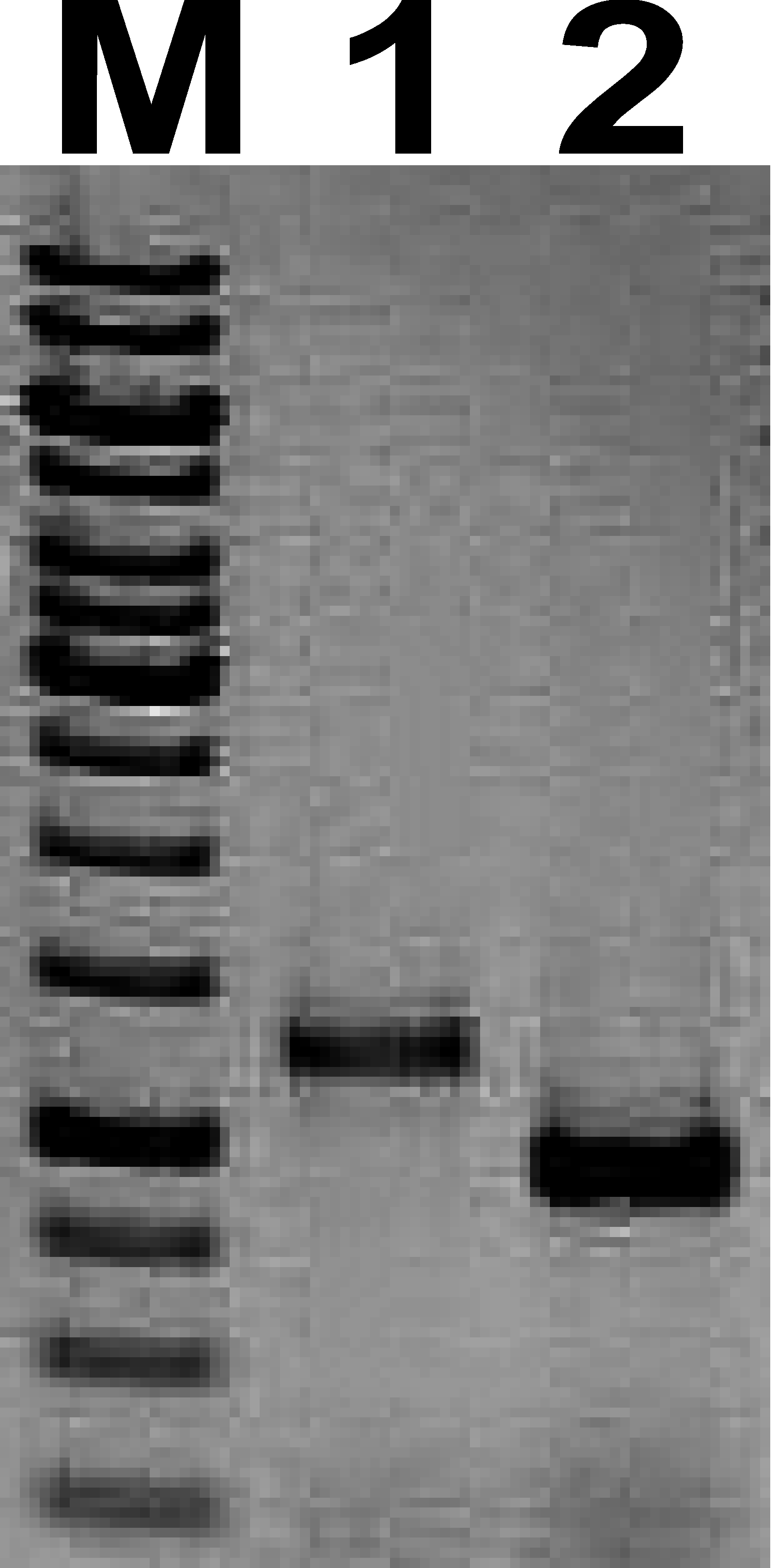

Supplement: S1 Fig — Agarose (1%) gel electrophoresis of PCR products (4 μl) visualized by staining with ethidium bromide is shown. M, 1 kb DNA Ladder (Thermo Scientific). The figure shows colony PCR of B. subtilis 168 (Lane 1) and BsΔyutF (Lane 2). DNA was amplified using primers BsC and (+)yutFs_PstI. (TIF) [file pone.0167580.s001.tif]

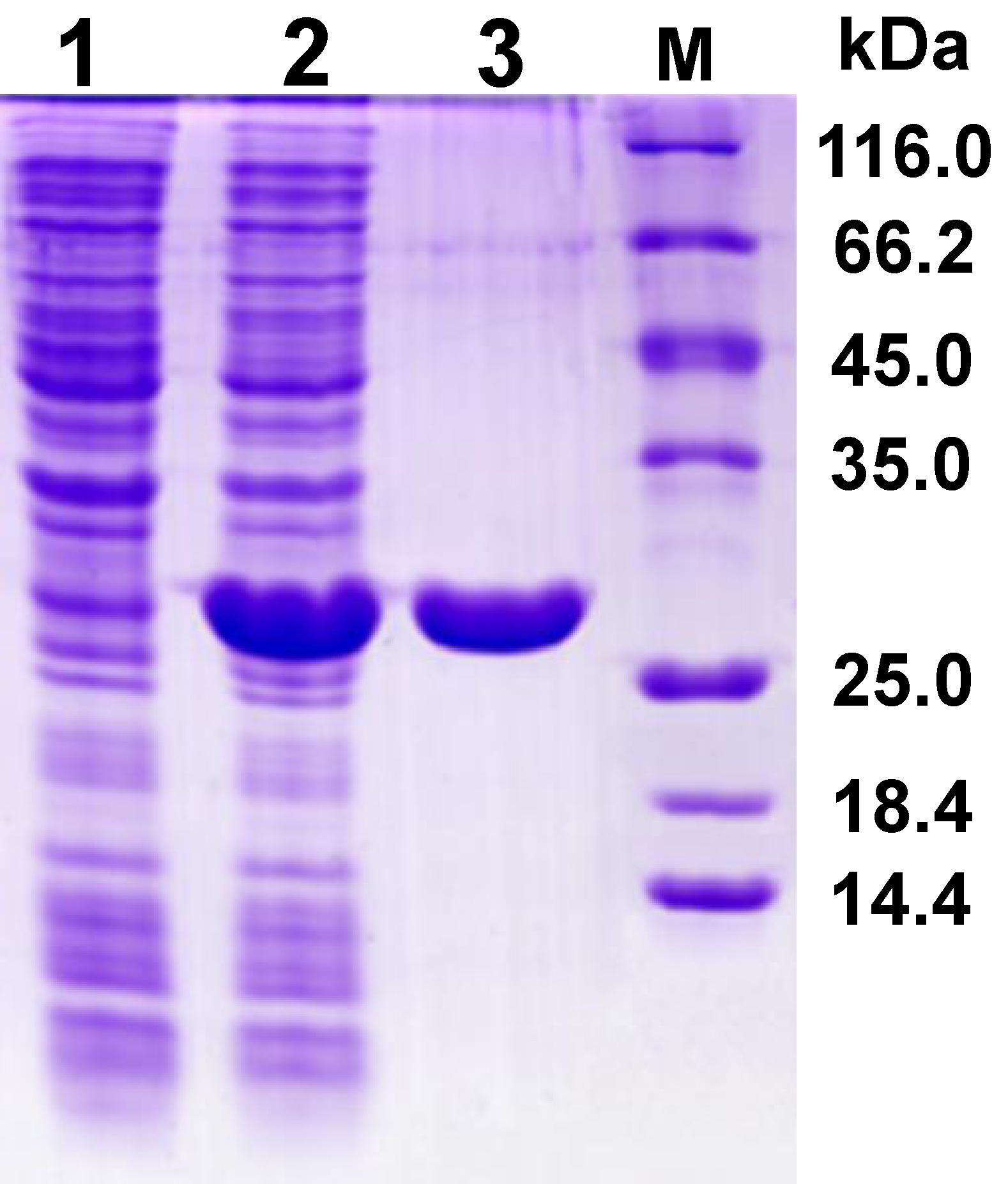

Supplement: S2 Fig — Lanes: 1, cellular lysate of BL21(DE3) harboring pET15b(+) induced with IPTG (17 μg of total protein); 2, cellular lysate of BL21(DE3) harboring pET15-H6-YutF induced with IPTG (17 μg of total protein); 3, the purified Ht-YutF product (5 μg). M, molecular mass standard (Unstained Protein Molecular Weight Marker, Thermo Scientific). Protein samples were separated by SDS-PAGE and stained with Coomassie Brilliant Blue. (TIF) [file pone.0167580.s002.tif]

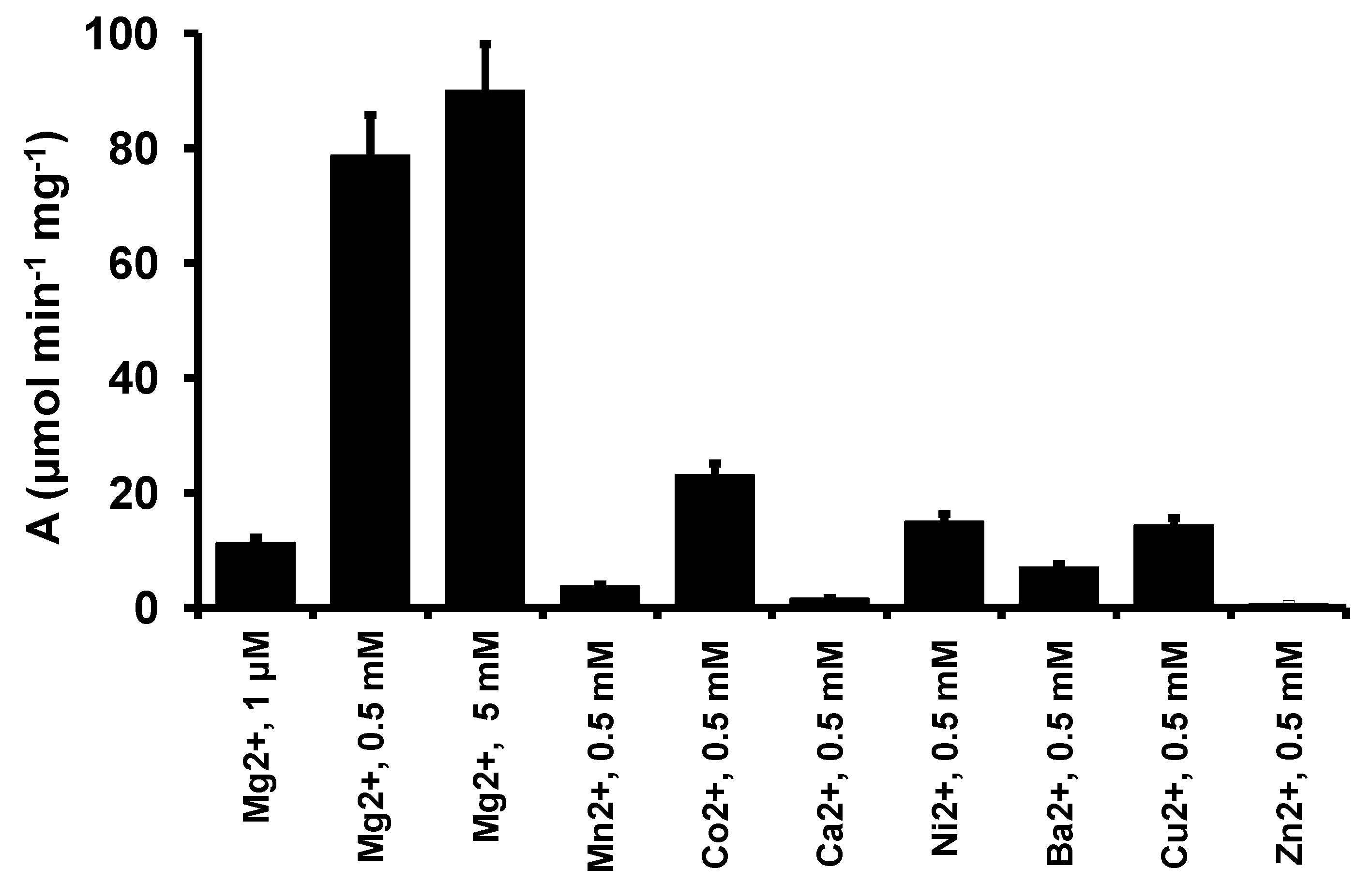

Supplement: S3 Fig — (TIF) [file pone.0167580.s003.tif]

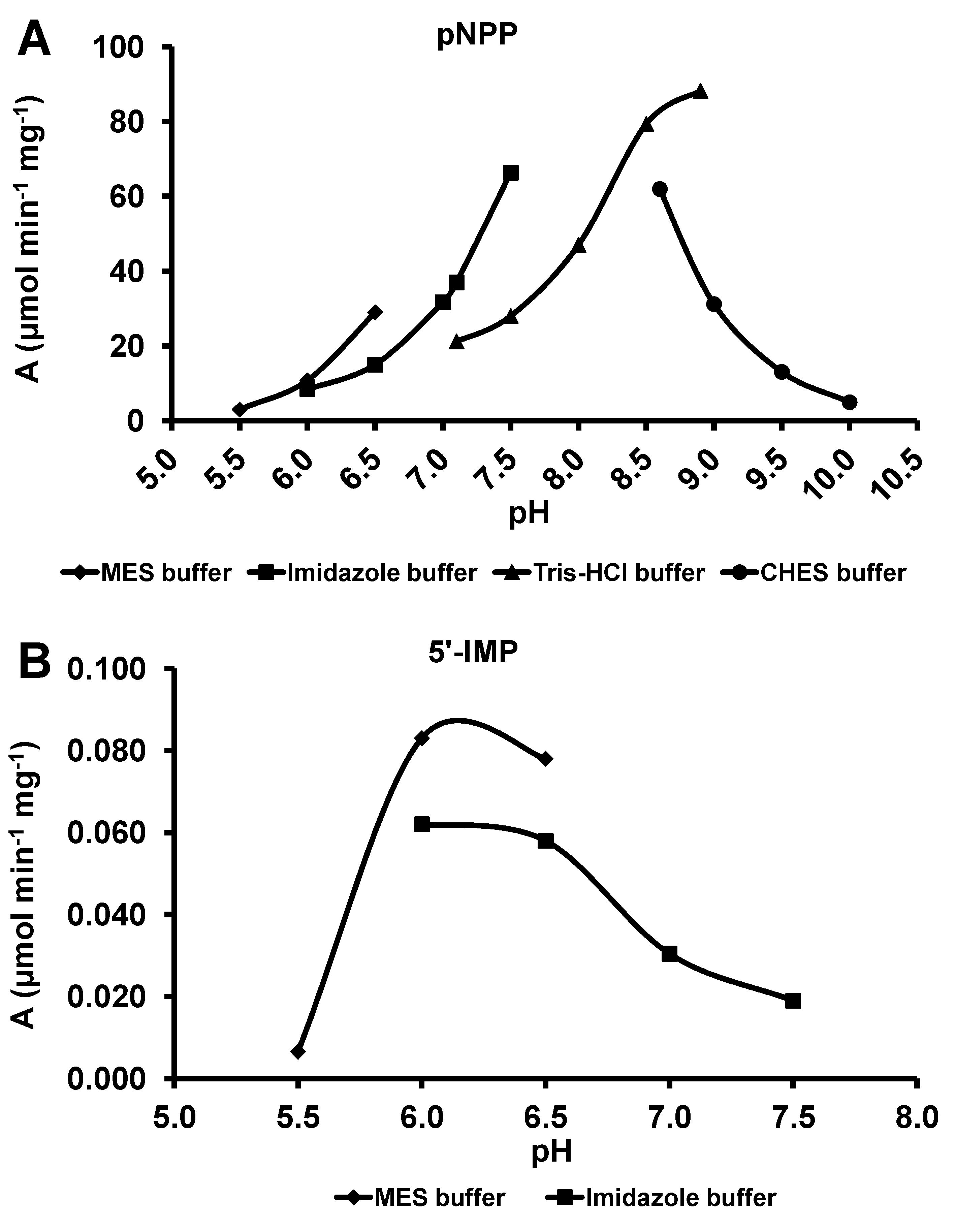

Supplement: S4 Fig — pH dependence of the phosphatase activity of purified Ht-YutF toward (A) pNPP (5 mM) and (B) 5'-IMP (5 mM). (TIF) [file pone.0167580.s004.tif]

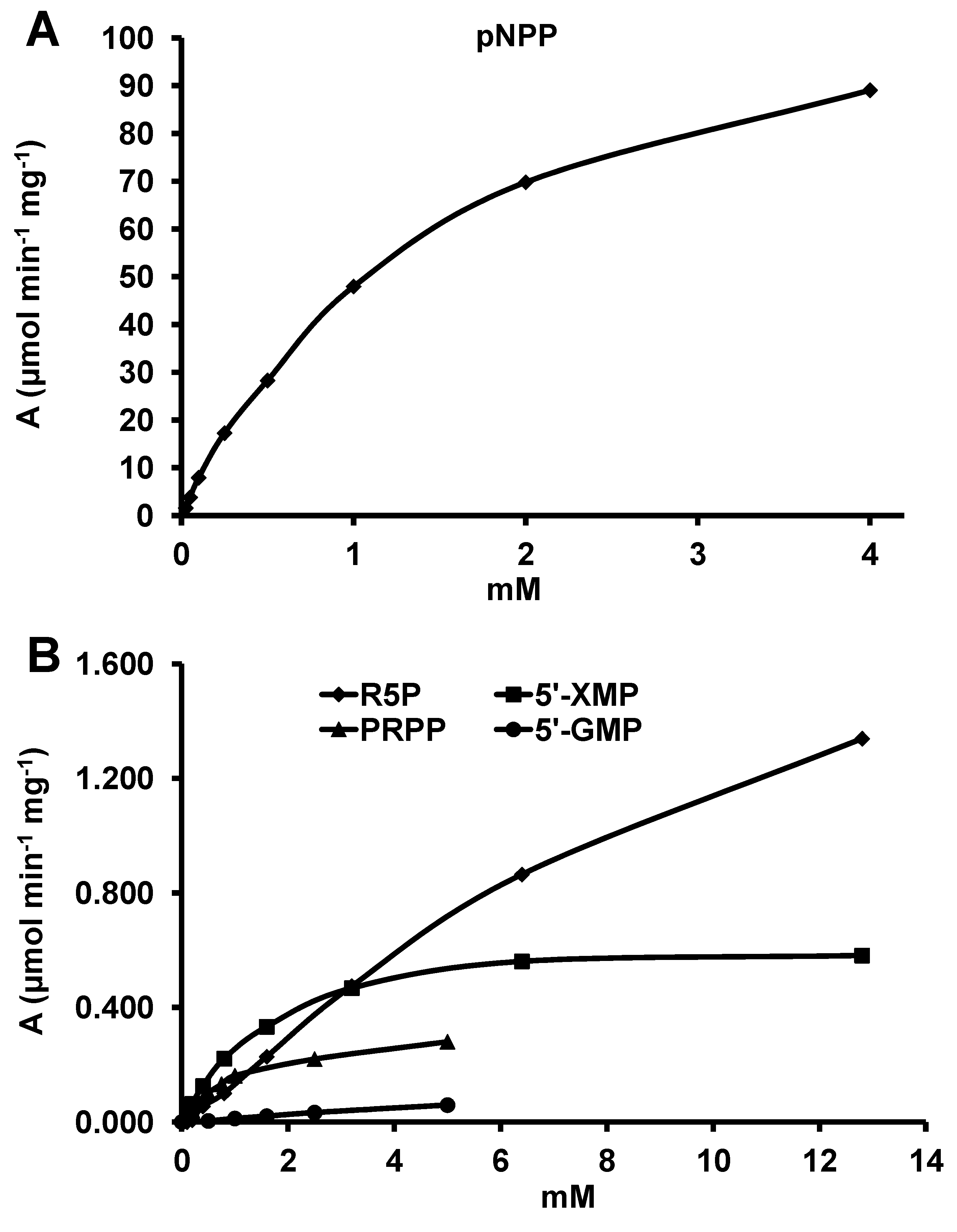

Supplement: S5 Fig — Substrate titration plots of Ht-YutF for (A) pNPP, (B) 5'-XMP, PRPP, R5P and 5'-GMP. (TIF) [file pone.0167580.s005.tif]
